# Study protocol: a lifestyle intervention for African American and Hispanic prostate cancer survivors on active surveillance and their partners

**DOI:** 10.1186/s40814-020-00653-7

**Published:** 2020-08-08

**Authors:** Dalnim Cho, Karen Basen-Engquist, Chiara Acquati, Hilary Ma, Curtis Pettaway, Yisheng Li, Cassandra S. Diep, Lorna H. McNeill

**Affiliations:** 1 Department of Health Disparities Research, UT Texas MD Anderson Cancer Center, 1400 Pressler St, 9th floor, Houston, TX 77030 USA; 2 Department of Behavioral Science, UT Texas MD Anderson Cancer Center, Houston, TX USA; 3 Graduate College of Social Work, University of Houston, Houston, TX USA; 4 Department of General Oncology, UT Texas MD Anderson Cancer Center, Houston, TX USA; 5 Department of Urology, UT Texas MD Anderson Cancer Center, Houston, TX USA; 6 Department of Biostatistics, UT Texas MD Anderson Cancer Center, Houston, TX USA; 7 Kinesiology Department, Rice University, Houston, TX USA

**Keywords:** Active surveillance, Black, Latino, Lifestyle behaviors, Physical activity, Healthy eating, Disparity, Cancer

## Abstract

**Background:**

Prostate cancer is the most commonly diagnosed cancer in both African American and Hispanic men. Active surveillance is a treatment option for low- or very low-risk prostate cancer survivors, and lifestyle interventions have been found to reduce the disease progression and improve the quality of life for both survivors and their partners. To date, no lifestyle interventions that specifically target African American or Hispanic men and their partners exist. This protocol describes a study that tests the feasibility of a randomized controlled trial, a lifestyle intervention developed to enhance healthy lifestyle and quality of life among African American and Hispanic men on active surveillance and their partners.

**Methods:**

A mixed-method study, including a two-arm randomized controlled trial (*n* = 30 dyads in the intervention arm and *n* = 10 dyads in the control arm) and in-depth interviews, will be conducted. Intervention arm participants will receive bi-weekly health coaching calls (a total of 12 calls based on Motivational Interviewing), as well as physical activity-specific (e.g., power point slides, print materials about physical activity, and activity trackers for self-monitoring) and nutrition-specific education (e.g., two nutrition counseling sessions from a registered dietitian, print materials about nutrition, and food intake recording for self-monitoring) over 6 months. All participants will be assessed at baseline, month 3, and month 6. Blood will be collected at baseline and month 6 from the prostate cancer survivors. Finally, in-depth interviews will be conducted with subsamples (up to *n* = 15 dyads in the intervention arm and up to *n* = 5 dyads in the control arm) at baseline and months 3 and 6 to conduct a process evaluation and further refine the intervention.

**Discussion:**

If effective, the intervention may have a higher health impact compared with a typical lifestyle intervention targeting only survivors (or partners), as it improves both survivors’ (tertiary prevention) and partners’ health (primary prevention). Results from this study will provide important information regarding recruiting racial/ethnic minority cancer survivors and their partners. Lessons learned from this study will be used to apply for a large-scale grant to test the impact of the dyadic intervention in a fully powered sample.

**Trial registration:**

ClinicalTrials.gov (NCT No. 03575832) registered on 3 July 2018.

## Background

Prostate cancer (PCa) is the most commonly diagnosed cancer in both African American (AA) [1] and Hispanic men [2]. PCa incidence and mortality rates are 1.7 and 2.4 times higher, respectively, among AA men than among non-Hispanic White (NHW) men [1]. Although Hispanic men report lower PCa incidence and mortality rates than NHW counterparts [2], significant variations in the mortality rates exist by country of origin: Mexican men have lower death rates than NHW men, but Hispanic men from Puerto Rico and Cuba have higher death rates than NHW men [2]. Furthermore, poorer quality of life (QoL) has been reported by AA and Hispanic PCa survivors compared with their NHW counterparts [3, 4].

Active surveillance (AS), in which survivors’ conditions are closely monitored, is a management option for low-risk or very low-risk PCa survivors; survivors proceed to treatments (e.g., surgery and radiation therapy) when their disease is clinically progressed. AS is a safe and feasible treatment plan [5–7]. In a study that followed 5302 AS men across 18 countries, PCa mortality was < 1% at both 5 and 10 years after diagnosis [7]. Another study found that after nearly 20 years of follow-up, surgery was not associated with significantly lower PCa mortality than AS, but it was associated with a higher frequency of long-term adverse events (e.g., impotence and incontinence) than AS [8]. PCa treatments lead to long-term side effects, such as urinary incontinence, erectile dysfunction, and fatigue, which may greatly impact not only PCa survivors’ but also their partners’ QoL [9, 10]. Thus, AS has the potential to reduce the risk of overtreatment of clinically low- or very low-risk PCa.

Beyond disease progression, however, psychological issues may play a critical role in the individual’s decision about AS. For instance, anxiety and uncertainty about disease progression can make PCa survivors opt out of AS and receive unnecessary treatments; anxiety was a significant predictor of treatment receipt among AS men, even when socio-demographics, baseline clinical characteristics, and Prostate-Specific Antigen (PSA) velocity were taken into account [11]. Although the percentage of those who decided to receive treatment due to anxiety varied across studies, it was as high as 13% [12].

To date, there are no established recommendations for delaying or preventing the progression of the disease in AS men [13]. However, several preliminary studies have shown that lifestyle interventions targeting diet and exercise are promising for reducing disease progression and improving QoL. For example, in a randomized controlled trial (RCT) in which 93 AS men were assigned to a 1-year intensive lifestyle intervention group addressing diet, exercise, and stress management or a usual care control group, results showed that 0% of the survivors in the intervention group received treatment, whereas 12% of the survivors in the control group received treatment [14]. In addition, serum PSA level decreased (4%) in those in the intervention group, whereas it increased (6%) in those in the control group. At the second year follow-up, 5% of the survivors in the intervention group received active treatment, compared with 27% of the patients in the control group [15]. Further, a single-arm lifestyle intervention with 31 AS men reported that psychological distress (intrusive thoughts and avoidance) regarding PCa decreased, and mental health-related QoL enhanced 3 months from the intervention [16]. Given these benefits, the National Institutes of Health has recommended more research on the development of lifestyle and therapeutic interventions for survivors undergoing AS [17].

However, none of the studies available in the literature has specifically targeted AA or Hispanic men on AS, who probably would benefit the most from the lifestyle changes. Also, none of the lifestyle interventions has included partners, who are likely to be the main source of support for PCa survivors [18, 19]. Overall, the inclusion of partners is supported by previous works showing that people are more likely to change their behaviors if their significant others are also motivated to change [20, 21]. In addition, as partners also experience insecurity and anxiety during AS [22], lifestyle interventions reducing partners’ anxieties may empower them to be more supportive of behavior change for the survivors. The inclusion of partners is also supported among racial/ethnic minorities, given that partners of AA men play significant roles (as counselor, coordinator, and confidant) in cancer-related decision-making [23], and Hispanics value familismo (commitment, loyalty, and dedication to family) and thus may want to include family members in the health decision-making process [24] or may defer decision-making to other family members [25].

To date, only two pilot physical activity interventions have been published that included both cancer survivors and their partners [26, 27], and these interventions seemed to be feasible (adherence and retention rates were > 70%, and no adverse effects were reported) and efficacious to enhance physical activity and QoL. Although these results are promising, only a few AA and Hispanic men were enrolled in those lifestyle interventions, and therefore, it is not clear whether lifestyle interventions will be feasible for AA and Hispanic men on AS. Taken together, it is warranted to develop and implement a culturally tailored intervention that enhances lifestyle behaviors and QoL and reduces anxiety in partners, as well as AA and Hispanic men on AS.

The present study will test a lifestyle intervention, *Watchful Living*, for AA and Hispanic men on AS and their partners. The primary aim of the study is to determine the feasibility of recruiting the target participants and implementing Watchful Living. Secondary aims are to (1) evaluate the preliminary efficacy of the intervention in improving diet, physical activity, partner’s support for lifestyle behaviors, QoL, and inflammation and (2) conduct a process evaluation of the intervention.

## Methods

### Design

**Fig. 1:**
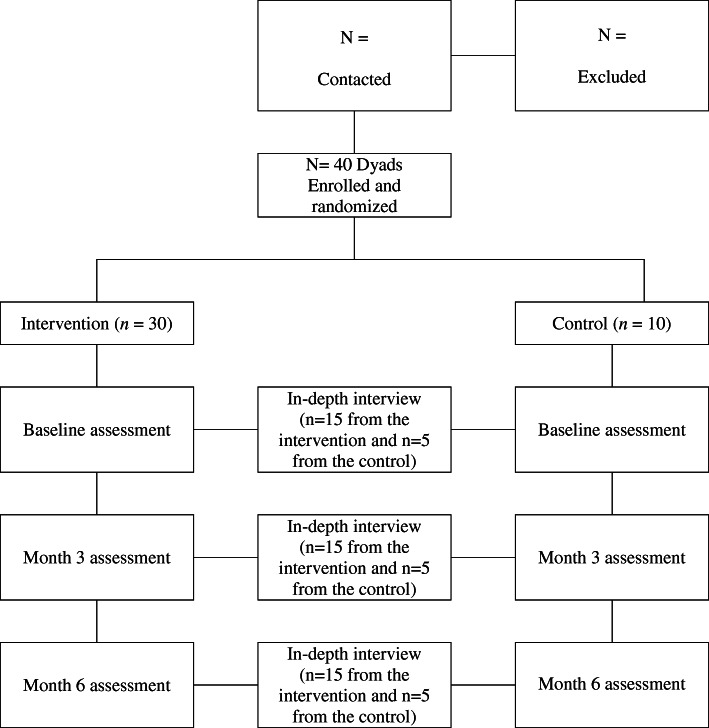
Participant flow

This is a mixed-methods study that incorporates a two-arm, pilot RCT and in-depth interviews. Specifically, a total of 40 survivor-partner dyads will be randomly assigned to either intervention arm or control arm. Participants will be assessed at baseline, month 3 (mid-assessment), and month 6 (post-intervention assessment). Dyads in the intervention arm will receive a home-based lifestyle intervention over 6 months. Those assigned to the control arm will receive a list of physical activity and healthy eating behavior resources (websites) at baseline and the same physical activity and nutrition materials that the intervention dyads receive after completing the month 6 assessment. In addition, 15 dyads from the intervention arm and five dyads from the control arm will be invited for in-depth interviews to conduct a process evaluation. The flow diagram of the trial is presented in Fig. 1.

### Recruitment and data collection procedures

A total of 40 dyads (i.e., 80 individuals) will be recruited and randomly assigned to either intervention (*n* = 30 dyads) or control arm (*n* = 10 dyads). Participants will mainly be recruited from the MD Anderson Cancer Center (MD Anderson) and Lyndon B. Johnson hospital in the Houston, TX area with collaborations with the Department of Urology at each site. Eligible participants will be retrieved from the hospital’s electronic health records. An invitation letter with urologists’ signatures will be sent to the survivors, and approximately 10 days to 2 weeks later, survivors will receive a call to be invited to the study. Study staff will explain the study, and only survivors who are interested will be screened for inclusion/exclusion criteria via telephone. To recruit partners, research staff will ask permission to contact partners via telephone to screen their eligibility. To facilitate enrollment of dyads, eligible survivors will be asked to come with their partners to MD Anderson. In particular, if survivors were treated at MD Anderson, our study visit will be scheduled on the same day as their follow-up appointment at MD Anderson. Thus, they will not need to come to MD Anderson again only to participate in our study. For all participants, we will cover their parking expenses.

In addition, we will recruit participants from the community with help from the Center for Community-Engaged Translational Research (CCETR; Director: McNeill). CCETR is an institutional research resource for investigators that maintains relationships with over 70 local and national organizations, providing a platform to assist with identifying and developing partnerships with Houston-area community organizations. For interested persons who contact us by telephone, hear a detailed description of the study, and provide oral informed consent to be screened, they will be screened for the inclusion/exclusion criteria. Those ineligible will be thanked for their time, and those eligible will be scheduled for an in-person baseline visit. As the participants will be recruited from multiple sites, block randomization will be used to ensure the same number of participants in each arm from each recruitment site.

All participants will be assessed at three time points: baseline, month 3, and month 6. Eligible dyads will attend an in-person baseline visit at MD Anderson. At this visit, study staff and research assistants will describe study procedures, complete consent forms, and collect baseline data. PCa survivors’ blood will be collected as well. Approximately a week later, the dyads in the intervention arm will receive an exercise prescription from an exercise physiologist and nutrition counseling from a registered dietitian in-person or via telephone. At month 3, the dyads will complete self-administered questionnaires at home, and blood will not be collected. At month 6, the dyads will visit MD Anderson and complete self-administered questionnaires. Blood draw again will be conducted among PCa survivors. Self-administered questionnaires will be completed either online (via RedCap) or on paper. For in-person data collection at baseline and month 6, survivors will receive a $25 gift card per visit, and partners will receive a $20 gift card per visit. For month 3, survivors and partners will receive a $15 gift card each. Participants will be anticipated to be enrolled in the study in July 2019.

### Participants

PCa survivors will be eligible if they (1) are AA or Hispanic men, (2) have clinically localized PCa diagnosed within 5 years prior to study enrollment, (3) are eligible to undergo AS or currently undergoing AS, (4) enroll with a spouse or intimate partner, (5) are ready and able to be physically active, as determined by a physician, (6) do not meet physical activity guidelines (i.e., 150 min of moderate physical activity or 75 min of vigorous physical activity), (7) have internet access at home (or at community center or church), and (8) have a valid home address and phone number.

PCa survivors will be excluded if they (1) have an active noncutaneous malignancy at any site; (2) had prior radiation therapy for treatment of the primary tumor; (3) have planned concomitant immunotherapy, hormonal therapy, chemotherapy, or radiation therapy during the study period; (4) participated in formative interviews for the study; and (5) participate in another physical activity, diet, or lifestyle program. Partners are eligible if they (1) are adults (≥ 18 years old) and (2) are ready and able to be physically active, as determined by a physician. The survivor-partner dyads can be married or unmarried, as well as same-sex or heterosexual. If one dyad member chooses to withdraw from the study, the other member will also be asked to withdraw from the study.

### Intervention

**Fig. 2:**
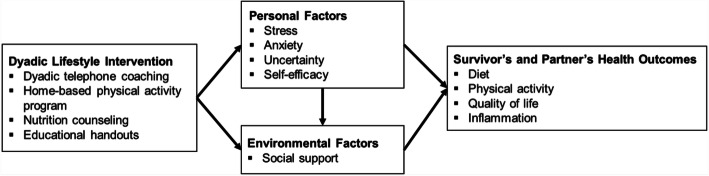
Conceptual model

A conceptual model that focuses on the role and influence of social relationships (i.e., the partner) will be used to guide this investigation (Fig. 2). There is no one theory that adequately explains the link between social relationships and health [28]. Although social cognitive theory [29] and social support theory have been used extensively for behavior change [30], these theories lack a focus on how individuals communicate with and influence one another. Interdependence theory is a dyad-level theory that emphasizes the outcomes experienced by both the agent and target of influence in a reciprocal fashion, by focusing on how influence and communication affect behavior [31, 32]. Therefore, the proposed model for this study is derived from these theories and is based on three key tenets: (1) social influence as a fundamental behavior change process, (2) interaction between personal factors (e.g., stress) and environmental factors (e.g., social support) to enhance behavior change, and (3) adequate levels of personal and environmental factors to initiate and maintain behavior change. We present format, goals, and behavior change techniques for each of our intervention components in Table 1.

**Table 1:** Intervention components, format, goals, and behavior change techniques

Intervention components	Format	Goals	Behavior change techniques
Health coaching	▪ Biweekly telephone counseling with a licensed counselor	Provide support for each other to meet the physical activity and healthy eating goals	▪ Motivational interviewing ▪ Provide information on consequences of behavior to the individual ▪ Goal-setting ▪ Prompt review of behavioral goals ▪ Social support ▪ General communication skills training
Home-based physical activity program	▪ Exercise prescription ▪ Printed materials ▪ Power point slides (voices over) ▪ Activity tracker	Engage in 150 min of moderate or vigorous physical activity per week	▪ Goal-setting ▪ Action planning ▪ Barrier identification/Problem solving ▪ Provide rewards ▪ Time management ▪ Self-monitoring of behavior ▪ Relapse prevention
Nutrition counseling	▪ Twice (in-person and/or via telephone) counseling sessions with a registered dietitian ▪ Printed materials (prevent T2) ▪ Food intake record (via MyFitnessPal.com or on paper)	Improve an overall diet quality including eat 5 servings of fruits and vegetables, reduce red meat intake, reduce sweetened beverage, and eat whole grain	▪ Goal setting ▪ Provide feedback on performance ▪ Provide instruction on how to perform the behavior ▪ Self-monitoring of behavior

#### Dyadic telephone coaching

The health coaching component will incorporate Motivational Interviewing and other motivation-based techniques to enhance commitment to and intrinsic motivation for diet and physical activity. The underlying goal is to evoke and support intrinsic motivation, which will lead dyads to initiate and persist in behavior change and maintenance efforts, as well as enhance retention in the program. The coaching will also employ cognitive-behavioral techniques to improve self-efficacy and other problem-solving and coping skills. Each dyad will receive 12 telephone coaching calls, each lasting approximately 45–60 min, from a master’s level trained counselor. Coaching calls will occur biweekly over the 6 months. During telephone coaching sessions, the health coach will ask about physical activity levels and eating behaviors, monitor the dyad’s progress, provide feedback, assist in solving physical activity and healthy eating barriers, address health concerns, and discuss goals until the next session. The survivor and partner will set their own behavior goals and begin at their own pace. They will also be encouraged to set a common behavior goal (either physical activity or healthy eating) that they engage in together. However, the health coach will emphasize that it does not mean that all activities should be done together and address how to support each other (e.g., providing emotional and practical support and avoiding nagging). All calls will include both members of the dyad, given the non-sensitive nature of the topics (e.g., behavioral skills, diet, and physical activity) and the focus on social support and interdependence.

#### Home-based physical activity program

The home-based physical activity program is adapted from an existing, evidence-based intervention, Active Living After Cancer (ALAC) program developed for cancer survivors [33]. The ALAC program is a theory-based, in-person and group-based program that consists of 12 weekly sessions that cover goal setting, benefits, and barriers to physical activity, problem solving, rewarding yourself, time management, self-efficacy, social support, and behavioral maintenance. The sessions are held in the community and led by trained health educators with PowerPoint slides. Participants receive a workbook with information on cognitive and behavioral skills to increase physical activity, written exercises to practice these skills, and case examples of cancer survivors who have successfully made physical activity changes. In addition, participants engage in 10–15 min activities together (e.g., resistance training and stretching and Zumba) and discuss survivorship topics (e.g., nutrition, fatigue and emotional distress). The goal of our home-based physical activity program is to engage in at least 150 min of moderate or vigorous physical activity per week. The program will provide the physical activity workbook and PowerPoint slides with our health coach’s voices over them. The workbook and PowerPoint slides will be slightly modified to target PCa survivors, couples, and racial/ethnic minorities. Furthermore, each member in the dyad will receive an individualized exercise prescription based on their current level of physical activity and health status from an exercise physiologist that includes options for activity selection (e.g., walking or bicycle riding), duration (time spent engaging in activity, ~ 20–60 min), frequency (number of days per week), and intensity (low and moderate). Finally, participants will receive an activity tracker to facilitate self-monitoring and a resistance training band.

#### Nutrition counseling

Each dyad will receive two nutrition counseling sessions with a registered dietitian. The first session occurs approximately a week after the baseline assessment (via telephone or in-person), with the second occurring at the time of the dyad’s choice before month 3. Participants will discuss goals, objectives, lifestyles, and current dietary habits with the dietitian. The goal is to get participants to improve diet, such as eating at least 2.5 cups of fruits and vegetables per day and reducing processed meat and red meat intake. Participants will be encouraged to record their food intake via MyFitnessPal.com or on paper, which we will provide in a binder, to encourage self-monitoring. Participants will also receive seven selected materials, including tracking food, healthy shopping and cooking, understanding carbohydrates, and eating well away from home, from prevent T2, a type 2 diabetes prevention program from the Centers for Disease Control and Prevention.

### Control group

The dyads assigned to the control arm will receive a list of physical activity and healthy eating behavior resources (websites) at baseline. In addition, after completing the month 6 assessment, they will receive the same physical activity and nutrition materials that the intervention dyads received.

### Measures

#### Lifestyle behaviors

Physical activity will be assessed with an accelerometer (ActiGraph wGT3X+ or wGT3X-BT), as well as with the International Physical Activity Questionnaire (IPAQ)-short form. Accelerometers capture overall body movement, intensity of activity, and time. Participants will wear small (pager-sized) blinded accelerometers (i.e., participants cannot see the values) just above their right hipbone for 7 days to assess typical physical activity in all forms (e.g., light, moderate, and vigorous activity). To be valid, the accelerometer should be worn a minimum of 10 hours per day [34] and for 5 days [35]. All participants will be given a self-addressed pre-paid envelope to return the accelerometer to the study staff after they have worn it for 7 days. For remote data collection at month 3, the device will be mailed to the participant, and a pre-paid envelope will be provided to mail back to study staff. For month 6, the accelerometer will be mailed 1 week before the visit, and the participant will be asked to return it at their in-person month 6 visit. The IPAQ assesses walking for exercise, walking for transportation, moderate and vigorous physical activity, and time spent sitting. It is widely used to measure physical activity and has shown good test-retest reliability (Spearman’s rho clustered 0.8 for all versions) and fair criterion validity (median rho = 0.30) [36]. Diet behaviors, including fruit and vegetable consumption and fat intake, will be assessed with a web-based tool, the Diet History Questionnaire-III [37]

#### Quality of life

The 12-item short form health survey (SF-12) is a widely used QoL measure producing two summary scores: physical component score and mental component score. The SF-12 has adequate validity and reliability and is positively associated with wellbeing [38].

#### Blood markers (survivors only)

Four inflammatory markers will be assessed using blood samples at baseline and month 6, as they are associated with PC patients’ mortality [39, 40]: C-reactive protein (CRP), tumor necrosis factor alpha (TNF-alpha), interferon gamma (IFN-gamma), and interleukin 4 (IL-4). CRP is selected because it is a global marker of inflammation and is predictive of chronic conditions, including cardiovascular disease [41]. TNF-alpha similarly indicates a systemic inflammatory state. IFN-gamma is chosen because it increases when there is a type I immune response (i.e., cell mediated or inflammatory), whereas IL-4 increases when there is a type II immune response (i.e., humoral or anti-inflammatory).

#### Anthropometric measures

At baseline and month 6, weight and height (to calculate body mass index), waist and hip circumference, and % of body fat will be assessed from research staff. Height (cm) will be measured without their shoes with a stadiometer, and weight (kg) and % body fat will be assessed with Tanita Body Fat Analyzer (TBF-300A). Waist and hip circumference (in) will be assessed with Myotape. Each of them will be measured twice. At month 3, self-reported weight and height will be collected.

#### Self-efficacy

Self-efficacy for exercise and healthy eating will be assessed with a total of 32 items [42]. Participants will use a 5-point Likert scale, from 1 (*I know I cannot*) to 5 (*I know I can*), to rate their level of confidence in being physically active (12 items) and reducing calories and fats (25 items). Both the exercise (*α* ≥ .83) and healthy eating items (*α* ≥ .85) have good internal consistencies.

#### Social support

Social support will be measured using items from the Social Support and Exercise Survey developed by Sallis to assess social support from family and friends [43] and later modified by Eyler [44] for use with racially/ethnically diverse populations. The items in the modified version will assess both emotional (three items, *α* = .77) and informational (three items, *α* = .71) social support using a 5-point Likert scale to measure participants’ agreement or disagreement from 1 (*strongly disagree*) to 5 (*strongly agree*).

#### Perceived stress

The Perceived Stress Scale [45] assesses the degree to which respondents find their lives to be stressful. This scale has been widely used in various cultures and countries for measuring psychological stress and has established acceptable psychometric properties.

#### Anxiety

Participants’ state anxiety will be assessed using the 6-item State Anxiety Scale [46]. The 6-item version has acceptable reliability and validity, producing similar scores to those obtained using the original version.

#### Uncertainty

Illness uncertainty will be measured using six items adapted from the Mishel Uncertainty in Illness Scale (MUIS) [47], which assesses survivors’ perceptions of uncertainty about their disease using a 5-point Likert scale from 1 (*strongly disagree*) to 5 (*strongly agree*). Also, we will use another six items adapted from the Assessment of Survivor Concerns (ASC) [48], whose convergent and discriminant validity was supported. Each item is asked to respond using a 4-point Likert scale from 1 (*not at all*) to 7 (*very much*). Partners will answer about their uncertainty regarding their partners’ PCa.

#### Dyadic adjustment

A 7-item version of the Dyadic Adjustment Scale (DAS-7), which assesses level of agreement on topics (e.g., philosophy of life, aims/goals/beliefs, and amount of time spent together), frequency of time together (e.g., working together on a project), and happiness, will be measured. The DAS-7 has a good internal consistency (Cronbach’s alpha ranged from .75 to .80) and demonstrated its discriminant and concurrent validity [49].

#### Communication

Communication about cancer will be assessed using items adapted from the Couple’s Illness Communication Scale (CICS) [50]. The CICS comprises four items for survivors (e.g., “It is hard for me to express feelings about my illness to my partner”) and partners (e.g., “It is hard for me to express feelings about his/her illness to my partner”) each. Participants are asked to respond using a 5-point Likert scale from 1 (*disagree strongly*) to 5 (*agree strongly*). We will modify “illness” to “prostate cancer.” The CICS has good internal consistency (.84 for patients and .80 for partners) and adequate test-retest reliability over a 3-month period (.71 for patients and .75 for partners).

#### Analytic plans

The intervention adherence rate for each dyad is defined as the proportion of intervention sessions completed by both the survivor and partner. We will look at percentage of both survivor and partner completing a minimum of 9 out of 12 coaching calls and both nutrition counseling sessions. Feasibility of recruitment will be achieved if ≥ 30% of eligible participants enroll in the study, and ≥ 80% participants complete follow-up assessments at months 3 and 6. Feasibility of intervention adherence will be achieved if average proportion of intervention adherence rate is ≥ 0.7 (e.g., coaching calls and nutrition sessions completed by both the survivor and partner ≥ 70%).

Linear mixed models will be used when combined data from both survivors and partners are analyzed due to their correlation within couples. The primary endpoint for the efficacy of the interventions is the differences between intervention and control group in outcome changes between baseline and 6 months post baseline. Specifically, an intervention indicator will be included and tested for the difference of increase in outcomes between groups. In our data, cluster corresponds to couples and random intercepts will be used to represent the pair effects. We will also conduct analysis of covariance to compare efficacy outcomes of interest pre and post intervention between intervention and control group separately for survivors and partners. Covariates and corresponding baseline outcome will be adjusted for in the analyses. Two-sided statistical tests will be performed, and the significance level for each test will be set at 0.05 without adjustment for multiple testing. All results from the statistical tests will be interpreted as hypothesis generating rather than conclusive findings. We will additionally perform various descriptive analyses, including dyadic analyses, to characterize the sample and compare participants and those who refused/withdrew on demographic and medical factors.

A process evaluation of the intervention will be conducted using in-depth interviews at each assessment time point with a subsample of the survivor-partner dyads (up to *n* = 15 dyads from the intervention arm and up to *n* = 5 dyads from the control arm). Questions will address participants’ experiences; thoughts about strengths, weaknesses, and needed improvements; perceptions about outcomes and impact; and overall reactions to and satisfaction with the intervention. Control arm dyads are also included in the interviews to understand their experiences and behavior changes over the study time period. This approach will assist us identifying elements of the intervention that were successful and/or need improvement for the larger trial. All interviews will last no longer than 2 h and will be audio-taped and transcribed verbatim. Transcripts will be entered into NVivo and analyzed using thematic data analysis procedures [51]. Two individuals will independently code the data for all transcripts to minimize potential biases.

### Sample Size Justification

Our sample size of 40 dyads provides adequate statistical support to help evaluate our primary aim of feasibility. In terms of evaluating feasibility based on recruitment, if the true consent rate of eligible dyads is 85%, then there will be 88% probability for us to screen 50 couples or less to arrive at 40 couples that consent; if the true consent rate is 75%, then there will be only 26% probability for us to screen 50 couples or less to arrive at 40 couples consenting. With the chosen total study sample size of 40 dyads, the randomization ratio would then be determined by the sample size chosen specifically for the intervention arm. With a lack of data available in the literature on the adherence of the proposed intervention among racial/ethnic minority PCa patients and partners, it would be valuable to allocate more patients to the intervention arm than the control arm to allow a more accurate assessment of the intervention adherence rate (as part of the primary objective of the study).

Specifically, when the sample size is 30, a two-sided 95% confidence interval (CI) for the average adherence rate will extend 0.36 standard deviation (SD) from the observed average rate, where SD is the estimated standard deviation of the adherence rate, assuming the CI is based on the large sample z statistic. This is in contrast to the corresponding half width of the 95% CI for the average adherence rate of 0.44 SD when the number of dyads in the intervention arm is 20 (as a result of a 1:1 randomization ratio), representing a less precise estimate of the mean adherence rate. Finally, we would like to keep a control arm with a reasonable number of patients (10 as chosen) to allow assessment of preliminary efficacy of the intervention with a reasonable level of accuracy, yet as a secondary objective of the study.

## Discussion

To our knowledge, Watchful Living will be the first dyadic lifestyle intervention conducted among AA and Hispanic men on AS and their partners. Strengths of this study will include (1) use of a mixed-methods research design, (2) application of the systematic intervention adaptation process, (3) assessment of inflammatory markers, and (4) use of an objective measure of physical activity. If effective, the intervention may have a higher health impact compared with a typical lifestyle intervention targeting only survivors (or partners), as survivors’ morbidity/mortality might be reduced (tertiary prevention), and partners’ own susceptibility to cancer may be decreased (primary prevention).

Recruiting cancer survivors is challenging, and recruiting couples will be an additional challenge. Given that numerous barriers have been reported with regard to participating in cancer research by underrepresented populations [52] and participating in health research by racial/ethnic minorities [53], various recruitment strategies (both passive and active) will be required. In particular, building strong team works with multiple hospitals and multidisciplinary teams including investigators who work with racial/ethnic minority cancer survivors and family members may be a key to succeed. We expect that results from this study will inform helpful strategies for recruitment and intervention with racial/ethnic minority PCa cancer survivors on AS and their partners. Lessons learned from this study will be used to further refine the intervention and apply for a large-scale grant.

## Data Availability

The datasets generated and/or analyzed during the current study are not publicly available due to confidentiality but may be available from the corresponding author on reasonable request.

## Abbreviations

PCa: Prostate cancer
AA: African American
NHW: Non-Hispanic White
QoL: Quality of life
AS: Active surveillance
PSA: Prostate-specific antigen
SF-12: 12-item short form health survey
CRP: C-reactive protein
TNF-alpha: Tumor necrosis factor alpha
IFN-gamma: Interferon gamma
IL-4: Interleukin 4
MUIS: Mishel Uncertainty in Illness Scale
ASC: Assessment of Survivor Concerns
DAS-7: Dyadic Adjustment Scale
CICS: The Couple’s Illness Communication Scale
CI: Confidence interval
SD: Standard deviation
